# Anabolic Steroids Activate the NF-κB Pathway in Porcine Ovarian Putative Stem Cells Independently of the ZIP-9 Receptor

**DOI:** 10.3390/ijms25052833

**Published:** 2024-02-29

**Authors:** Kamil Wartalski, Jerzy Wiater, Patrycja Maciak, Agnieszka Pastuła, Grzegorz J. Lis, Marcin Samiec, Monika Trzcińska, Małgorzata Duda

**Affiliations:** 1 Department of Histology, Jagiellonian University Medical College, Kopernika 7 Street, 31-034 Krakow, Poland; kamil.wartalski@uj.edu.pl (K.W.); jerzy.wiater@uj.edu.pl (J.W.); grzegorz.lis@uj.edu.pl (G.J.L.); 2 Department of Endocrinology, Institute of Zoology and Biomedical Research, Faculty of Biology, Jagiellonian University in Krakow, Gronostajowa 9 Street, 30-387 Krakow, Poland; patrycja.maciak@doctoral.uj.edu.pl; 3 Department of Genetics and Evolutionism, Institute of Zoology and Biomedical Research, Faculty of Biology, Jagiellonian University in Krakow, Gronostajowa 9 Street, 30-387 Krakow, Poland; agnieszka.pastula@uj.edu.pl; 4 Department of Reproductive Biotechnology and Cryoconservation, National Research Institute of Animal Production, Krakowska 1 Street, 32-083 Balice, Poland; monika.trzcinska@iz.edu.pl

**Keywords:** pig, ovary, putative stem cells, NF-κB signalling pathway, ZIP-9, boldenone, nandrolone

## Abstract

Boldenone (Bdn) and nandrolone (Ndn) are anabolic androgenic steroids (AASs) that, as our previous studies have shown, may increase the risk of neoplastic transformation of porcine ovarian putative stem cells (poPSCs). The NF-κB pathway may be important in the processes of carcinogenesis and tumour progression. Therefore, in this work, we decided to test the hypothesis of whether Bdn and Ndn can activate the NF-κB pathway by acting through the membrane androgen receptor ZIP-9. For this purpose, the expression profiles of both genes involved in the NF-κB pathway and the gene coding for the ZIP-9 receptor were checked. The expression and localization of proteins of this pathway in poPSCs were also examined. Additionally, the expression of the ZIP-9 receptor and the concentration of the NF-κB1 and 2 protein complex were determined. Activation of the NF-κB pathway was primarily confirmed by an increase in the relative abundances of phosphorylated forms of RelA protein and IκBα inhibitor. Reduced quantitative profiles pinpointed not only for genes representing this pathway but also for unphosphorylated proteins, and, simultaneously, decreased concentration of the NF-κB1 and 2 complex may indicate post-activation silencing by negative feedback. However, the remarkably and sustainably diminished expression levels noticed for the *SLC39A9* gene and ZIP-9 protein suggest that this receptor does not play an important role in the regulation of the NF-κB pathway.

## 1. Introduction

Nuclear transcription factors κB (NF-κB) are a family of related proteins involved in the regulation of many significant genes. The NF-κB family was first described in the nuclei of murine B lymphocytes by Ranjan Sen and David Baltimore from the Massachusetts Institute of Technology [1]. In mammals, the existence of five NF-κB family proteins (NF-κB1 p105→p50, NF-κB2 p100→p52, RelA, RelB, and c-Rel), which are encoded by five corresponding genes, has been proven so far. Importantly, proteins are further divided into two classes that share common structural features [2,3]. Rel class proteins contain a conserved C-terminal transactivation (TAD) domain in their structure, through which they can activate transcription. However, NF-κB class proteins do not include the TAD domain and function as transcriptional repressors [4]. Proteins from the NF-κB family form homo- and heterodimers, but only heterodimers (e.g., p50/RelA or p52/RelB) act as transcription factors. Moreover, the activity of heterodimeric protein pairs is tightly regulated by their binding to such inhibitors of the IκB protein family as IκBα, IκBβ, IκBε, IκBζ, and Bcl-3 [5,6]. Attachment of the IκB inhibitor to the NF-κB/Rel complex masks the nuclear localization signal (NLS) sequence in the NF-κB subunit, preventing the translocation of the NF-κB/Rel dimer to the cell nucleus [7].

The direct activation of the NF-κB/Rel complex is mediated by IKK (inhibitor of nuclear factor-κB) kinases responsible for the phosphorylation of IκB and NF-κB2 [8]. Indirectly, there are at least three ways of NF-κB activation, namely: classical, alternative, and atypical. The factors causing activation include numerous groups of compounds, such as pro-inflammatory cytokines, bacterial lipopolysaccharides, viral proteins, and mitogens. The influence of oxidative and genotoxic stresses is also extremely important, especially in atypical pathways [9,10,11,12]. In cells, the expression level and activity of the NF-κB transcription factor must be precisely regulated by negative and positive feedback. However, numerous possible disorders in signal transduction in the NF-κB pathway and its constitutive activation are some of the causes of progression in cancer transformation [13]. Moreover, NF-κB induces the expression of many anti-apoptotic genes (e.g., *CFLAR*, *BIRC2*), which seems to support the point of view that cancer cells can avoid apoptosis by using this signalling pathway [14,15].

Anabolic androgenic steroids (AASs) are substances produced from testosterone or its chemical derivatives. These compounds exhibit anabolic or androgenic properties, depending on their destination in tissues and organs [16,17]. AASs can bind to the androgen receptors (ARs) with high affinity. Acting as both agonists and antagonists of ARs, AASs modify endogenous hormone levels and the availability of specific receptors [18]. The illegal use of AASs is very popular among athletes, bodybuilders, and youth because of their anabolic features and improving physical condition. These compounds are also commonly used as feed additives for farm animals, and their metabolites contaminate the environment through urine. It is worth emphasizing that the International Agency for Research on Cancer included AASs in the group of potentially carcinogenic chemicals for humans (IARC Group 2A) because these compounds may have both cytotoxic and genotoxic effects (dependent on NF-κB atypical pathways), which may result in neoplastic transformation [19]. The most frequently chosen AASs are popular drugs often used illegally in humans and animals, including boldenone (Bdn) and nandrolone (Ndn). Chemically, Bdn is a dehydrated analogue of testosterone and a good AR agonist [20,21]. The benefits for which Bdn is abused encompass, first and foremost, an increase in the protein synthesis and stimulation of the kidneys to release erythropoietin and intensify nitrogen retention [22]. Among all injectable AASs, Ndn is the most commonly taken or abused by representatives of different age groups within human populations worldwide [23]. Ndn, exerting its impacts through ARs, has a weak androgenic activity and, simultaneously, plays strong anabolic and progestogenic functions [20]. There are scientific reports confirming that Ndn negatively affects the physiology and functioning of the female reproductive system [24,25,26,27]. Ndn in physiological doses of this drug inhibits respiratory chain complexes (e.g., I, III) and mitochondrial respiration. Moreover, Ndn increases the production of mitochondrial reactive oxygen species (ROS) and also slows cell growth. Finally, noteworthy is the fact that Ndn continuously contributes to the maintenance of adult stem cells (ASCs) in the niches of various tissues, but, on the other hand, it may increase the risk of carcinogenesis [28,29].

Three main groups of stem cells (SCs) can be distinguished depending on their developmental stages: embryonic stem cells (ESCs), fetal stem cells (FSCs), and postnatal adult stem cells (ASCs). Fundamentally, ESCs have practically endless potential, but their clinical usage is strongly limited for reasons such as ethical issues, immunogenicity, and teratoma formation can followed by the development of malignant tumours (cancerous neoplasms) [30,31]. Currently, there is an increase in interest in ASC biology. Until recently, it was thought that differentiation of ASCs restricts their potential to be unipotent and limited to one tissue or organ. Nevertheless, there is growing evidence that ASCs have greater developmental plasticity than previously assumed. Therefore, it is real that they are even multipotent [32,33,34]. The ASCs have been shown to exist in most mammalian tissues and organs, including, inter alia, bone marrow, brain, heart, intestine, peripheral blood, skeletal muscle, skin, teeth, and testis [35]. They are localized in the special histological compartments and anatomotopographical areas of the above-indicated tissues and organs, respectively. These compartments/areas are designated as stem cell niches [36]. Not only scientific reports from the last few years but also research carried out by our team have confirmed the existence of ASCs in mammalian ovaries (including domestic pigs). Due to the large diversity of ovarian ASCs, they are also called putative stem cells (PSCs) [37,38,39]. The results of our previous studies [37,40,41] have demonstrated that porcine ovarian putative stem cells (poPSCs) isolated from the ovarian cortex (against the presence of SSEA-4 antigen), though the non-specific expression of selected pluripotency markers are not pluripotent but rather multipotent. We have shown that poPSCs can differentiate into functional neural-like and endothelial cells, poPSCs likely represent just multipotent mesenchymal stem cell (MSCs) populations in the ovary. This fact is evidenced by the expression of MSC-related markers such as *ITGB1* (CD29), *THY1* (CD90) and *ENG* (CD105), which is exhibited in poPSCs [41]. Additionally, in our latest report, we have proved that poPSCs display the expression of ARs by which AASs, including Ndn and Bdn, can influence them [29]. In the formerly cited scientific work, constant exposure of poPSCs to therapeutic doses of both Bdn and Ndn has been demonstrated to trigger alterations in the expression of such surface glycoproteins as CD44 and CD133. The presence of the above-mentioned clusters of differentiation indicates the threat of the occurrence of cytochemical events characteristic of the carcinogenesis of poPSCs. An increased expression of CD44 and CD133 following Bdn and Ndn exposure is proof of a phenotype shift from poPSCs, perhaps even to cancer stem cells (CSCs). This is solid support for the current hypothesis that suggests that tumours originate from cells that have undergone the process of “malignant reprogramming” as a result of genetic and/or epigenetic changes [42].

The existence of ARs in many types of stem cells (ESCs, MSCs, CSCs), including poPSCs, is common [29,43,44,45]. Furthermore, in 2014, a new membrane androgen receptor (mAR) was described in the ovarian cells of Atlantic Croaker. This mAR was identified as ZIP-9 (Zrt- and Irt-like protein 9), which is a member of the zinc transporter family and is encoded by the *SLC39A9* gene. What is important is that in humans, the expression of ZIP-9 has been found in breast and prostate cancer and affects testosterone-dependent apoptosis in ovarian follicles and cancer cells [46,47]. Converse and Thomas demonstrated that testosterone mediates not only pro-apoptotic but also anti-apoptotic responses in granulosa/theca cells in a follicle stage-dependent manner [48]. ZIP-9 is the only steroid receptor discovered to date that regulates zinc homeostasis through regulating zinc transport. ZIP-9 is also the only known member of the ZIP family that has been demonstrated to signal via G proteins [49]. ZIP-9 may also mediate non-classical testosterone pathways in Sertoli cells by increasing cAMP, activating ERK1/2 kinase signalling, and activating transcription factors such as ATF-1 and CREB [50]. It is worth highlighting that the role of androgens is diverse and not limited only to the reproductive systems. Androgens have been reported to influence, among others, the proliferation of cardiovascular endothelial cells. In this system, ZIP-9 mediates, e.g., androgen-induced early proliferation in human umbilical vein endothelial cells (HUVEC) [51].

Due to the above-mentioned premises, our research aims to check whether AASs represented by Bdn and Ndn can activate the NF-κB signalling pathway in poPSCs. For this reason, the pivotal goal of the present investigation is preponderantly confined to precisely finding the answer to our paramount question: if these selected anabolic steroids activate the intracellular NF-κB-dependent networks focused on inter-transcriptomic and inter-proteomic communication, would they affect poPSCs via the membrane androgen receptor ZIP-9? This report is a subsequent continuation of our recent study targeted at exploring the effect of AASs (Bnd and Ndn) on the potential change in the poPSCs phenotype to a CSCs-like one [29]. The change in the phenotype of poPSCs resulting from the plasticity of these cells is probably possible due to the activation of intracellular signal transduction pathways, including NF-κB. In the current work, we demonstrate that AASs can activate the NF-κB-related pathway in poPSCs by indirectly affecting the phosphorylation of key proteins such as IκBα and RelA. However, ZIP-9 does not appear to play a significant role in this signalling pathway.

## 2. Results

### 2.1. Exposure of poPSCs to Bdn or Ndn Has Impact Not Only on the Proteomic Profiles of NF-kB Pathway Representatives and ZIP-9 Receptor but Also on the Levels of Phosphorylated Forms of IκBα and RelA Proteins

**Figure 1:**
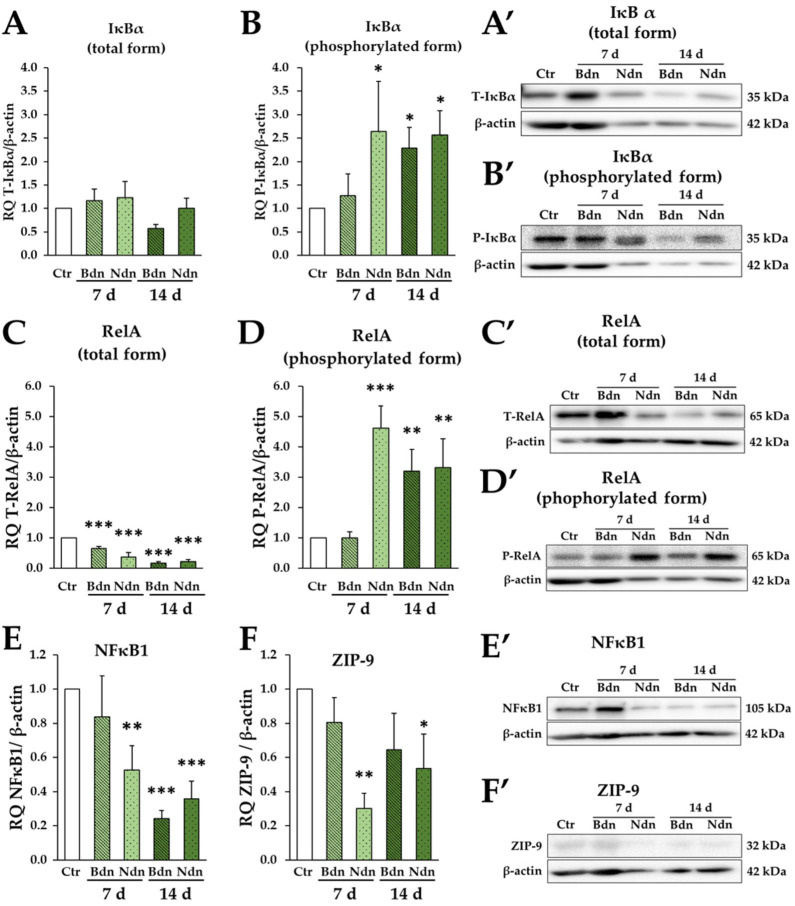
Expression of the NF-κB pathway proteins and the ZIP-9 receptor: total form of IκBα (**A**,**A′**), phosphorylated form of IκBα (**B**,**B′**), total form of RelA (**C**,**C′**), phosphorylated form of RelA (**D**,**D′**), NF-kB1 subunit 105 (**E**,**E′**) and ZIP-9 (**F**,**F′**) at the level of total protein on Days 7 and 14 of culture in the presence of boldenone (Bdn) or nandrolone (Ndn). The graphs show the relative expression of IκBα, RelA, NF-κB1, and ZIP-9 proteins obtained from measurements of the optical density of the bands representing a specific signal. Results represent the mean with *n* = 3 ± standard deviation (SD). Statistical analysis: homogeneity of variance—Levene’s test, normality of distribution—Shapiro–Wilk test, one-way ANOVA, Dunnett post-hoc test, * *p* ≤ 0.05; ** *p* ≤ 0.01; *** *p* ≤ 0.001.

In poPSCs exposed to boldenone (Bdn)- or nandrolone (Ndn)-mediated supplementation, after 7 and 14 days of treatment, fluctuations in the protein expression of IκBα inhibitors were observed. However, the observed changes were not significantly different from IκBα abundance in the poPSCs cultured without steroid supplementation, which served as a control (Figure 1A,A′). Importantly, in the same set of experiments, Bdn and Ndn usually caused a statistically significant, more than two-fold increase in the expression of the phosphorylated form of the IκBα inhibitor. Only in poPSCs cultured for 7 days in the presence of Bdn, statistically insignificant increases of the IκBα inhibitor were observed (Figure 1B,B′). Regarding the RelA protein, which is a key subunit of the p50/p65 dimer with transcriptional activity, a statistically significant decrease in the expression of the total form of RelA protein was observed in all trials of poPSCs cultured with anabolic androgenic steroids (AASs) (Figure 1C,C′). Analogously to the expression of the phosphorylated form of IκBα, the expression of the phosphorylated RelA protein increased significantly several times in all AAS-treated samples, except for a short 7-day exposure of poPSCs to boldenone (Figure 1D,D′). NF-κB1 as the p105 subunit is both an inhibitor of the NF-κB pathway and a precursor of the p50 subunit formed as a result of co-translational processing. The p50 subunit together with the p65 (RelA) subunit can form a transcriptionally active dimer. The AASs reduced the abundance of the NF-κB1 precursor protein in poPSCs. The strongest inhibition of NF-κB1 expression was demonstrated in poPSC-derived trials after 14 days of exposure to Bdn or Ndn. The weakest and statistically insignificant effect was exerted by Bdn during the 7-day exposure (Figure 1E,E′). Based on the presented results, it can be concluded that selected AAS may influence the activation of the NF-κB pathway through increased expression of phosphorylated IκBα and RelA proteins compared to their total forms. The decrease in the amount of NF-κB1 protein during the activation of the NF-κB pathway by steroids can be indirectly explained by the transformation of p105 subunits into p50. The present research showed that, although individual AASs are similar compounds, the pace of their action may be different. Nnd significantly influenced the expression of NF-κB pathway proteins both during 7 and 14 days of exposure. Meanwhile, 7-day exposure to Bdn usually did not significantly affect changes in protein expression in poPSCs. However, a significant effect of Bdn was already noticeable after 14 days.

The research hypothesis assumed that AASs may act on poPSCs through the membrane androgen receptor ZIP-9. Nevertheless, no increased expression of this receptor was observed in poPSCs treated with each representative of AASs (either Bdn or Ndn). Quite the opposite, in poPSCs cultured in the presence of Bdn or Ndn, decreases in ZIP-9 expression were observed compared to the control (cultures without the addition of steroids). However, only nandrolone had a statistically significant effect on poPSCs and significantly reduced the expression of ZIP-9 in their cell membranes (Figure 1F,F′).

### 2.2. Immunofluorescent Localization of NF-κB Pathway Proteins in poPSCs Cultured in the Presence of Ndn or Bdn

**Figure 2:**
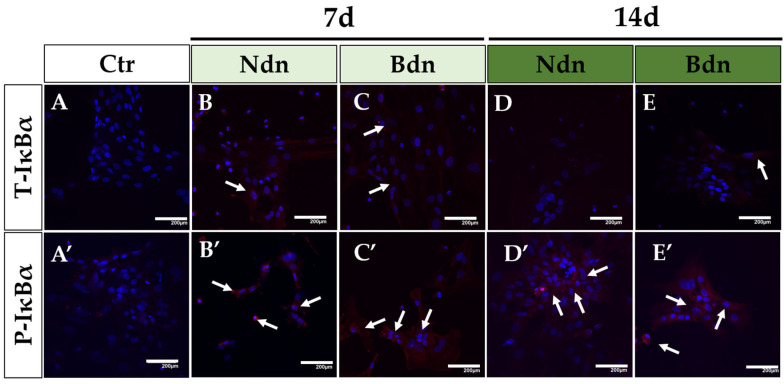
The presence and specific cytoplasmic localization of T-IκBα (**A**–**E**) and P-IκBα (**A′**–**E**′) in poPSCs cultured without the addition of anabolic steroids (**A**,**A′**) and in poPSCs cultured in the presence of nandrolone (Ndn; **B**,**B′**,**D**,**D′**) or boldenone (Bdn; **C**,**C′**,**E**,**E′**) for 7 and 14 days. Red signals—mediated by DyLight 594 fluorescent dye and derived from T-IκBα and P-IκBα proteins (immunofluorescent signals marked by white arrows), blue signals—dependent on DAPI and originating from the tagged DNA molecules within the cell nuclei; scale bars represent 200 μm.

The research was carried out on poPSCs cultured in the presence of Ndn or Bdn for 7 and 14 days. The derived poPSCs line served as a control trial. Only in some samples of poPSCs treated with each representative of AASs, i.e., either Ndn or Bdn (white arrows on Figure 2B,C,E), a weak immunofluorescent signal indicating the presence of the total form of IκBα inhibitor was observed. However, in the same set of experiments, the immunofluorescent signal derived from the phosphorylated form of the IκBα inhibitor was much more pronounced and observed in all the experimental samples (white arrows in Figure 2B′–E′). The weak immunofluorescence signal of the total form of IκBα and the more prominent signal of the phosphorylated form of IkBα were consistent with the results of IκBα expression analyses performed using the Western Blot method (Figure 1A,B). It is worth noting that both forms of the IκBα inhibitor exhibited specific cytoplasmic localization in poPSCs.

**Figure 3:**
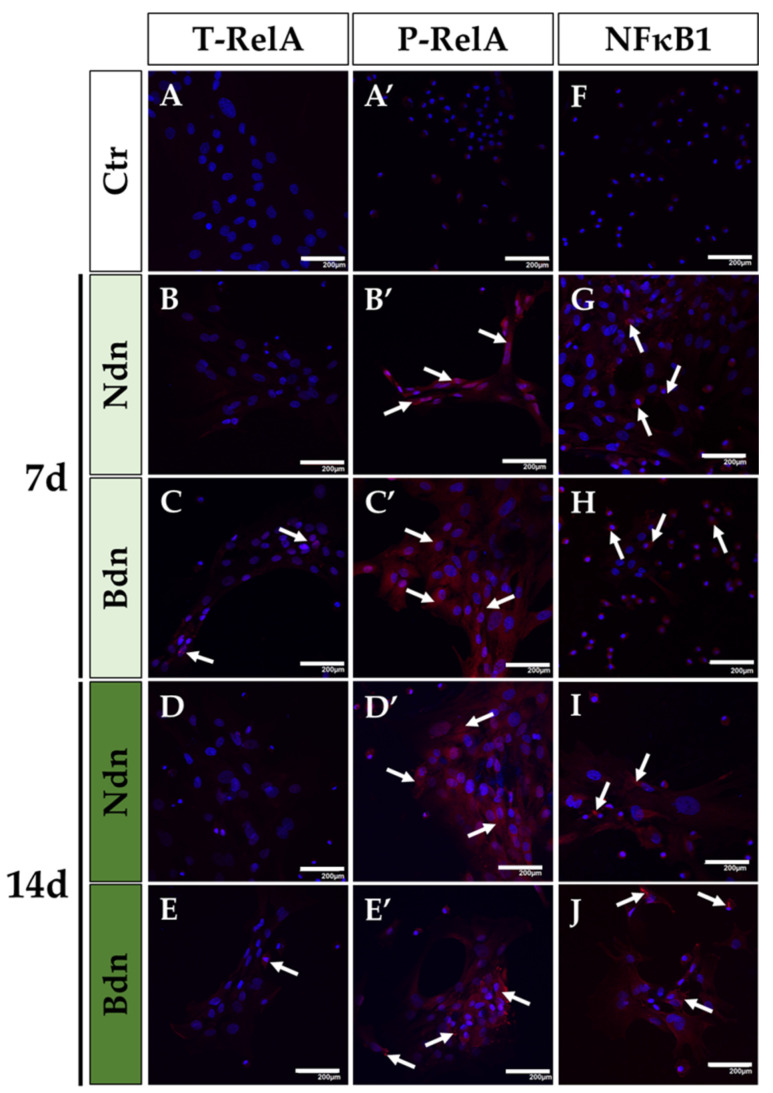
The presence and specific cytoplasmic localization of T-RelA (**A**–**E**), P-RelA (**A′**–**E′**), and NF-κB1 (**F**–**J**) in poPSCs cultured without the addition of anabolic steroids (**A**,**A′**,**F**) and in poPSCs cultured for 7 (**B**,**B′**,**G**,**C**,**C′**,**H**) and 14 days (**D**,**D′**,**I**,**E**,**E′**,**J**) in the presence of nandrolone (Ndn; **B**,**B′**,**G**,**D**,**D′**,**I**) or boldenone (Bdn; **C**,**C′**,**H**,**E**,**E′**,**J**). Red signals—mediated by DyLight 594 fluorescent dye and derived from T-RelA, P-RelA and NF-κB1 proteins (immunofluorescent signals marked by white arrows), blue signals—dependent on DAPI and originating from the tagged DNA molecules within the cell nuclei; scale bars represent 200 μm.

Immunofluorescent localization of RelA protein in poPSCs confirmed the results of RelA expression analysis demonstrated by Western blot. The signal originating from the total form of the RelA protein in poPSCs treated with each representative of AASs (either Ndn or Bdn) was trace/negligible and has been confirmed only in the 7- and 14-day tests dependent on boldenone treatment (white arrows in Figure 3C,E). Meanwhile, the signal from the phosphorylated form of RelA protein was specified; it was proven to be clear and strong in the cytoplasm of poPSCs cultured in the presence of either boldenone or nandrolone (white arrows in Figure 3B′–E′). Signal amplification from the phosphorylated form of RealA indicates increased activation of the NF-κB signalling pathway influenced by AASs. The relatively weak cytoplasmic signal from NF-κB1 was also observed in all groups of poPSCs cultured in the presence of Ndn or Bdn (white arrows in Figure 3G–J). In all immunofluorescence analyses performed on poPSCs treated with steroids, the most spectacular images concerned the phosphorylated forms of IκBα and RelA proteins. This confirms the hypothesis that AASs stimulate the NF-κB pathway via phosphorylation.

### 2.3. The Effects of Bdn or Ndn Treatments on the Concentration of NF-κB Pathway Proteins in poPSCs Cultured in the Presence of Selected AASs for 7 and 14 Days

**Figure 4:**
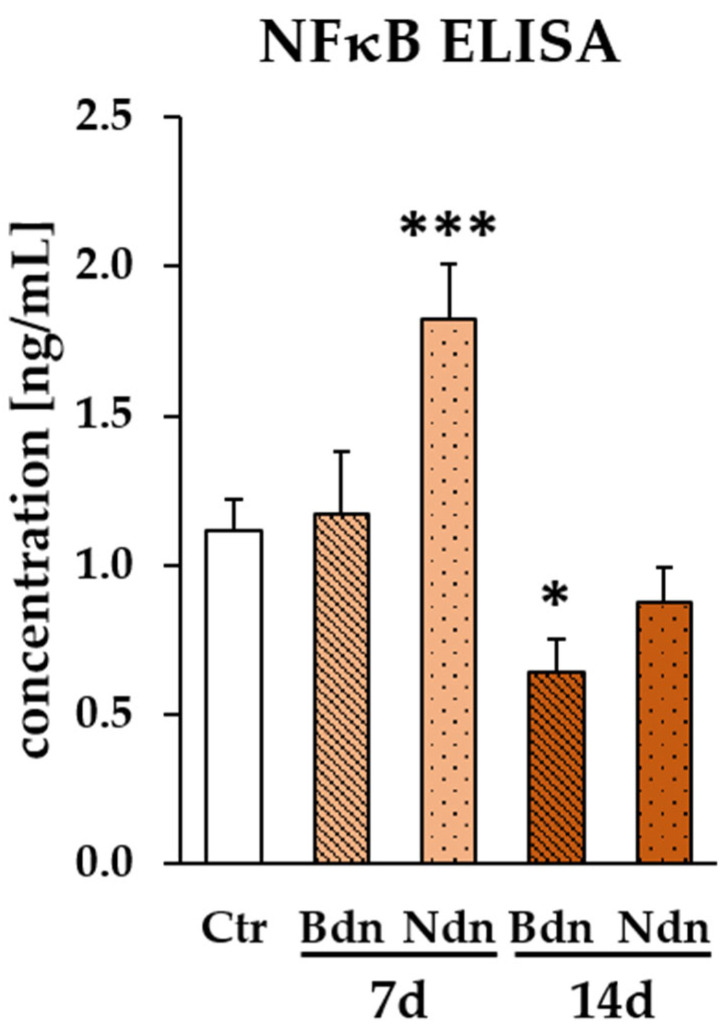
Concentrations of NF-κB pathway proteins (NF-κB1 and NF-κB2 complex) [ng/mL] in poPSCs lysates were assessed by ELISA as described in Materials and Methods. The results of each treatment were expressed as the fold change between control and boldenone (Bdn)- or nandrolone (Ndn)-treated cells. Data are expressed as the mean with *n* = 3 ± standard deviation (SD). Statistical analysis: homogeneity of variance—Levene’s test, normality of distribution—Shapiro–Wilk test, one-way ANOVA, Dunnett post-hoc test, * *p* < 0.05; *** *p* < 0.001.

The concentration of NF-κB pathway proteins (NF-κB1 and NF-κB2 complex) in lysates of poPSCs treated with Bdn and Ndn was determined by ELISA. Quantitative analysis of protein levels revealed a higher concentration of pathway proteins after a 7-day exposure to Bdn and a distinctly, almost twice higher concentration after a 7-day exposure to Ndn (*** *p* ≤ 0.001) in the samples of poPSCs tested in comparison to the control (culture of poPSCs without the addition of AASs). However, already 14 days of exposure to anabolic steroids resulted in a decrease in the concentration of NF-kB pathway proteins in the tested samples of PSCs as compared to the control group. It is worth noting that only boldenone caused a significant decrease (* *p* ≤ 0.05) in the concentrations of NF-κB pathway proteins (Figure 4). Decreasing concentrations of NF-kB pathway proteins in cell lysates observed with increasing exposure time to steroids may indirectly indicate the activation of a regulatory mechanism based on negative feedback. This is even more visible at the level of gene expression.

### 2.4. Analysis of the Expression Profiles for NFKBIA, RELA, NFKB1, and SLC39A9 Genes in poPSCs Treated with AASs for 7 and 14 Days

**Figure 5:**
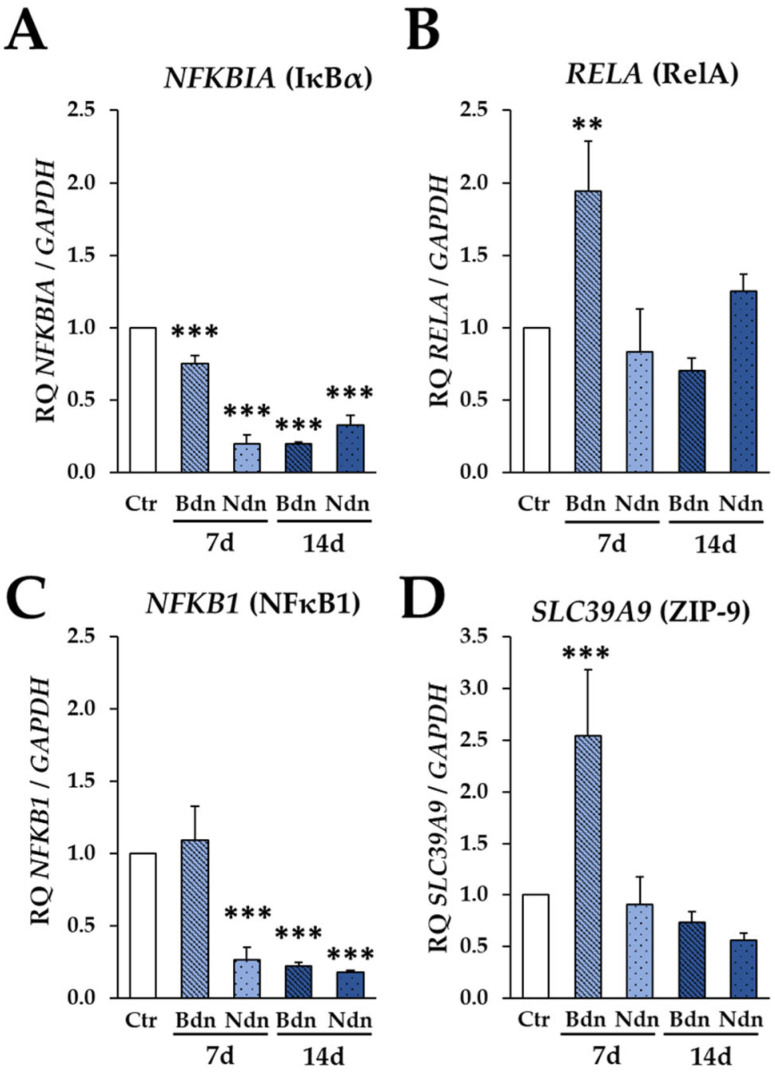
Expression of genes for NF-kB: *NFKBIA* (**A**), *NFKB1* (**C**), and *RELA* (**B**) and also for ZIP-9 receptor: *SLC39A9* (**D**) at 7th and 14th day of culture in the presence of boldenone (Bdn) and nandrolone (Ndn) versus poPSCs cultured without the addition of steroids at the transcript level as shown by RT-qPCR. The results (2^−ΔΔCt^) are presented as mean values with *n* = 3 ± standard deviation (SD). Statistical analysis: homogeneity of variance—Levene’s test, normality of distribution—Shapiro–Wilk test, one-way ANOVA and Dunnett post-hoc test, ** *p* ≤ 0.01; *** *p* ≤ 0.001.

Analysis of both the genes involved in the NF-kB signalling pathway (*NFKBIA*, *RELA*, *NFKB1*) and the gene coding for the ZIP-9 receptor (*SLC39A9*) by real-time PCR usually showed their significant downregulation in both groups of poPSCs treated either with Bdn or with Ndn, after 7 or 14 days of culture. *NFKBIA* mRNA expression in poPSCs expanded ex vivo for 7 and 14 days in the presence of Bdn and Ndn was significantly lower as compared to control cultures (*** *p* ≤ 0.001) (Figure 5A). *RELA* mRNA expression in poPSCs cultured for 7 days in the presence of Bdn was significantly higher compared to control cultures (** *p* ≤ 0.01). The expression of *RELA* mRNA in poPSCs cultured for 7 days in the presence of Ndn and for 14 days in the presence of Bdn or Ndn fluctuated compared to control cultures, but these changes were not statistically significant (Figure 5B). *NFKB1* mRNA expression in poPSCs cultured for 7 and 14 days in the presence of Bdn and Ndn was significantly lower as compared to control cultures (*** *p* ≤ 0.001) (Figure 5C). For *SLC39A9* mRNA, the quantitative profile of its expression in poPSCs cultured for 7 days in the presence of Bdn was significantly higher as compared to control cultures (*** *p* ≤ 0.001). However, the expression of *SLC39A9* mRNA in poPSCs cultured for 7 days in the presence of Ndn and for 14 days in the presence of Bdn or Ndn was lower as compared to control cultures but not statistically significant (Figure 5D). Decreasing levels of *NFKBIA* and *NFKB1* gene expression, and *RELA* expression oscillating close to that of the control sample are results that are often opposite to those obtained by Western blot. However, it is likely that the higher expression of NF-κB pathway proteins, especially the phosphorylated forms of IκBα and RelA, contributes to the silencing of their gene expression by negative feedback. *SLC39A9* gene expression confirms previous results at the protein level that the ZIP-9 receptor has a limited impact on the NF-κB pathway.

## 3. Discussion

Our results indicate that the anabolic androgenic steroids that we tested can activate the NF-κB pathway in porcine putative stem cells (poPSCs) derived from the ovarian cortex. The activation of this pathway is evidenced by the phosphorylation under the influence of AASs (mainly Ndn) of both the IκBα inhibitor and the RelA protein, which is part of the transcription factor. However, results such as the cytoplasmic (instead of nuclear) localization of NF-κB pathway proteins in poPSCs, oscillatory fluctuations in their concentrations, and the usually reduced expression of genes of NF-κB signalling pathway after exposure to steroids may indicate post-activation inhibition of the pathway by negative feedback. Moreover, our studies prove that signalling through the ZIP-9 receptor is essential in the activation of the NF-κB pathway.

Few studies have evaluated the effects of steroids on the NF-κB pathway. Studies on the toxic effects of AASs on the liver have shown that the NF-κB pathway can also be activated by steroids in a manner independent of oxidative stress and thus plays a protective role in counteracting the pro-apoptotic effect of AASs on hepatocytes [52,53]. Studies in rats have shown that resistance training combined with testosterone intake increases the expression of the NF-κB pathway and cyclooxygenase 2 (COX-2) genes, related to inflammation in the kidneys [54]. Furthermore, the NF-κB pathway can be activated by boldenone-induced oxidative stress, leading to the transcription of pro-inflammatory genes and thus the accumulation of pro-inflammatory factors such as TNFα and IL-1β [55,56]. Other studies assessed the effect of supraphysiological doses of nandrolone on oxidative stress and apoptosis in the brains of rats. Research has confirmed that nandrolone generates significant oxidative stress in various areas of the brain. Furthermore, the NF-κB pathway can be activated by reactive oxygen species resulting from the action of nandrolone. However, endogenous ROS in limited doses can only activate the NF-κB signalling pathway, and above a certain level, they can negatively impact this signalling [57]. Our studies provide evidence that boldenone and nandrolone can activate the NF-κB pathway, which is largely regulated by negative feedback loops, which is also indirectly confirmed by our analyses. Several regulatory loops are involved in the activation of the NF-κB pathway, acting mainly as negative feedback. The IκBα inhibitor is one of the main proteins in the intracellular negative feedback loop. The IκBα is encoded by the *NFKBIA* gene, which belongs to the so-called early genes activated by NF-κB. Activation of the NF-κB pathway leads to the expression of the IκBα protein and, subsequently, to the accumulation of the IκBα inhibitor in the cell. The IκBα binds to the NF-κB1 factor present in the cell nucleus, leading to its movement from the nucleus to the cytoplasm and therefore causing its inactivation [58]. The expression of A20/TNIP2 (TNF-Interacting Protein 2) proteins creates a second negative feedback loop by regulating the ubiquitination of adapter proteins and thus inhibiting the activation of the IKK kinase complex and also inhibiting further activation of the NF-κB pathway [59]. When the signalling pathway is constantly activated, the recently synthesized NF-κB-binding IκBα in the cytoplasm may also be phosphorylated by IKK, leading to the repetition of a series of biochemical events in the form of a loop. This leads to subsequent activation and inhibition of the NF-κB pathway combined with the relocation of protein factors (e.g., NF-κB1) between the cell nucleus and the cytoplasm. This phenomenon is sometimes called cytoplasmic-nuclear oscillations [60]. Importantly, also in the context of this work, long-term elevated NF-κB levels lead to chronic inflammation and counteract the mechanisms of programmed cell death, including those transformed into cancer, because NF-κB is a transcription factor for anti-apoptotic genes. Our results indicate that the addition of Bdn or Ndn in the culture medium may lead to cyclical/oscillatory activation of the NF-κB pathway in poPSCs. Activation of the NF-κB pathway is evidenced by the increased expression of both the phosphorylated form of the RelA and the phosphorylated form of the IκBα inhibitor, the phosphorylation of which leads to ubiquitination and proteasomal degradation [58]. The activation of the NF-κB pathway may also be indirectly started by reduced expression of the NF-κB1 protein, because it may indicate the processing of this precursor protein into the mature form of the p50 subunit. The RelA protein, together with the p50 subunit (the 50 kDa subunit is derived from the 105 kDa NF-κB1 subunit), forms a dimer that is a transcription factor. However, the cytoplasmic rather than nuclear localisation of the phosphorylated form of the RelA protein and the cytoplasmic localisation of the NF-κB1 precursor protein indicate that we are not yet dealing with a functional transcription factor. The reduced expression of the *NFKBIA* gene probably results from the feedback mechanism described above. The results indicate that there is activation of the NF-κB pathway through phosphorylation of RelA and IκBα. However, at this stage, there is no active transcription factor yet, so transcription of the *NFKBIA* gene is limited. Reduced expression of the *NFKB1* gene and NF-κB1 protein can also be explained by completed transcription and translation, and the resulting NF-κB1 precursor protein after processing forms the p50 subunit, which is necessary for the generation of a transcriptionally active dimer with RelA. A similar molecular scenario involves the concentration of the complex of the precursor proteins NF-κB1 and NF-κB2. After a shorter exposure of poPSCs to AASs, an increase in the concentration of these proteins was observed and the effect of Ndn was stronger. However, after longer exposure to AASs, a slight, but still significant, decrease in the concentration of complex proteins took place. Nonetheless, this diminishment has been found to undergo considerable intensification in the case of Bdn treatment. These decreases may be caused by the transformation of precursor proteins (NF-κB1 and NF-κB2) into target forms (p50 and p52) as a result of the activation of the NF-κB pathway. The fact that AASs can influence poPSCs through ARs was confirmed in our previous work. We showed that both poPSCs cultured without steroids and their cell counterparts cultured in the presence of Bdn or Ndn exhibited relatively constant expression of the AR receptor and its specific nuclear localization [29]. Therefore, in the current research, we decided to check whether AASs can also act through other ARs, such as ZIP-9 receptors belonging to mARs. The role of ZIP-9 in cancer and its potential interactions with signalling pathways, including NF-κB, are still poorly understood. Gou et al. [61] demonstrated that mRNA levels for the ZIP-9 receptor were significantly reduced in human and mouse hepatocellular carcinoma. Furthermore, the removal of the ZIP-9 receptor specifically reduced the polarisation of M2 macrophages through the IL-4/STAT6 signalling pathways, but in turn, increased the polarisation of M1 macrophages through the NF-κB signalling pathway in response to inflammatory factors. In the NF-κB pathway, ZIP-9 specifically inhibits M1 macrophage polarization by reducing the phosphorylation of IκBα and IκBβ inhibitors [61]. Testosterone has a high affinity for ZIP-9 and acts as an agonist of this receptor, but other androgens, such as androstenedione and dihydrotestosterone, have a rather low affinity for ZIP-9 [49]. Perhaps this is one of the reasons for the low expression of both the *SLC39A9* gene and the ZIP-9 receptor protein in our studies. Boldenone and nandrolone displayed a significant affinity for the AR receptor, but, analogously to androstenedione and dihydrotestosterone, they may have a weak affinity for ZIP-9. From the cytophysiological point of view, ZIP-9 is tremendously interesting because it plays a dual role. It is both a zinc (Zn^2+^) transporter and an mAR receptor. Zinc cations are a key element necessary for the construction of many proteins, including zinc finger-containing transcription factors and metalloenzymes, as well as several signalling pathways related to cell proliferation, growth, and apoptosis. Zinc homeostasis is essential for human health. Therefore, disturbances in zinc regulation are associated with many pathologies, including diabetes, inflammation, and the development of cancer [62]. Strong expression of the ZIP-9 receptor and androgen binding have been identified, among others, in ovarian cancer [47]. However, both our previous studies [21] and the present one indicate that, although the morphology (differentiation and hypertrophy) and the phenotype (CD44 and CD133 positive) of poPSCs change under the influence of Bdn and Ndn, they are still not cells after neoplastic transformation, but in most cells with characteristics of precancerous lesions. Therefore, there may also be a weaker expression of the ZIP-9 receptor in poPSCs. Our research is the first report on the activation of the NF-κB pathway by Bdn and Ndn and through the ZIP-9 receptor.

In the future, to more precisely check how AASs influence the activation (maybe also inhibition) of the NF-κB pathway, the classical and alternative activation pathways of this pathway should be examined not only after the 7th and 14th days of culture with AASs but also at more frequent time points. Furthermore, it may be worth blocking classical nuclear ARs with a representative of non-steroidal anti-androgen agents, e.g., flutamide [63], to explore whether ZIP-9 receptors may play a greater role in signalling as a substitute for AR.

## 4. Materials and Methods

### 4.1. Collection of Porcine Ovaries and Subsequent Isolation of poPSCs

Porcine ovaries were obtained from sexually immature Polish Landrace gilts (approximately weighing 60–70 kg and 5–6 months of age). Tissues were obtained from commercially slaughtered pigs in a local slaughterhouse under veterinarian control within 10 min after slaughter. Then, samples were placed in sterile ice-cold Dulbecco’s modified phosphate-buffered saline (DPBS; pH 7.4, PAA The Cell Culture Company, Piscataway, NJ, USA) with the addition of antibiotics/antimycotic cocktail (Antibiotic/Antimycotic Solution; AASoln; 1% (*v*/*v*), PAA The Cell Culture Company, Piscataway, NJ, USA) and taken to the laboratory within 1 h. Following several-step washing of the experimental material with sterile DPBS, the ovarian cortex was separated from the ovarian cord with a scalpel and cut into uniform-size pieces of ~1 mm^3^ with a tissue slicer. The obtained fragments of ovarian cortex were subjected to a 2-h enzymatic digestion procedure in a Liberase™ TH Research Grade solution (0.26 U/mL in PBS; Sigma-Aldrich, Merck, St. Louis, MO, USA) in an incubator at 37 °C with 150 rotations/min. In the next step, enzymatic digestion was terminated by adding an equal volume of cold DPBS (+4 °C). After that, the resulting suspension was filtered through 100-, 70-, and 40-µm nylon cell strainers. The cells were subsequently washed several times in sterile DPBS and recovered by centrifugation (90× *g* for 10 min). poPSCs were isolated by applying a modified approach to the magnetic-activated cell sorting method, which had been elaborated and optimized in the previous investigation by Wartalski et al. [37]. In this approach, a monoclonal antibody—human anti-SSEA-4, which was conjugated/anchored to magnetic beads (EasySepTM hESC/hiPSC SSEA-4 Positive Selection Kit, StemCellTM Technologies, Vancouver, BC, Canada)—was used [37]. Afterwards, the poPSCs were cultured in the maintenance medium (MM), which was comprised of DMEM/F12 medium (Sigma-Aldrich, Merck, St. Louis, MO, USA) enriched with 2% B-27 (Thermo Fisher Scientific, Waltham, MA, USA) and 2 μL/mL SCF (Thermo Fisher Scientific Inc., Rockford, IL, USA). The prepared suspension of 3 × 10^3^ cells/mL was seeded into the culture dishes. To isolate the total protein or total RNA (following finalization of the experiment), the cells were cultured in six-well polystyrene plates (Nunc™, Thermo Fisher Scientific) coated with poly-l-lysine (Sigma-Aldrich, Merck). In turn, the ex vivo-expanded cells that originated from control groups intended for immunofluorescence studies were cultured on eight-cell Lab-TekTM II-CC2 (Nunc™, Thermo Fisher Scientific Inc.) slides also coated with poly-l-lysine.

### 4.2. Culture of poPSCs in the Presence of Selected Doses of Nandrolone or Boldenone

The evaluation of poPSC proliferation after 14-day exposure to different doses of nandrolone or boldenone was performed and thoroughly described in our previous study [29]. After preculture, the medium was replaced with its fresh counterpart (DMEM/F12, 2% B-27, 2 µL/mL SCF) and divided into two equal subgroups. The first subgroup was given a boldenone solution in DMSO so as to obtain a concentration of 100 μM in the medium, and the second subgroup received a nandrolone solution to obtain a concentration of 35 μM in the medium. The Ndn and Bdn concentrations for the experiment were selected based on both literature data and the results of the previous proliferation test. Every two days, the medium was changed, maintaining the dosing pattern of both test compounds, and the cells were passaged when they reached 80% confluence. After completion of culture on days 7 and 14, total protein and total RNA were isolated from cells growing in 6-well plates, and cells growing on eight-chamber slides were fixed for immunofluorescence.

### 4.3. Immunofluorocytochemistry-Based Analyses

Immunofluorescence, performed according to a technique developed and modified in our laboratory [29], was used to localize: NF-κB p65 phosphorylated form, NF-κB p65 total form, NF-κB p105, IKBα phosphorylated form, as well as IKBα total form in poPSCs cultured with the presence of steroids (boldenone and nandrolone). Additionally, PSCs cultured without the addition of steroids were used as a control. After culture termination, cells were washed with PBS and fixed with cold 4% paraformaldehyde (PFA) in PBS for 10 min. After several washes with PBS, permeabilization of the cell membranes was performed by applying 0.1% Triton X-100 (Sigma-Aldrich, Merck) in PBS. Then, nonspecific binding sites were blocked by incubation with 5% normal goat serum (NGS; Sigma-Aldrich, Merck) in a humidified chamber for 40 min at room temperature (RT). Next, the blocking solution was removed, and the cells were incubated overnight at 4 °C in a humidified chamber with the appropriate primary antibodies (see Table 1, ICC dilution in PBST). Subsequently, the cells were washed several times with PBST (PBS with 0.1% Tween 20; Sigma-Aldrich, Merck) and incubated with the goat anti-Rabbit DyLight594-conjugated secondary antibody (Abcam, Cambridge, UK) for 1 h at RT in a dark, humidified chamber. In the negative control, incubation with primary antibodies was omitted. Immuno-labelled cells were mounted in VectaShield^®^ HardSet™ Mounting Medium with DAPI (Vector Laboratories, Burlingame, CA, USA), and they were analysed with an OLYMPUS FV1200 FLUOVIEW scanning confocal laser microscope (Olympus, Tokyo, Japan) using 405 nm and 543 nm diode lasers for excitation DAPI and DyLight594 respectively, as well as both 20× and 40× objective lenses.

### 4.4. Western Blot Analysis

Western blot analysis was performed according to a technique developed and modified in our laboratory [41]. Briefly, after the termination of both poPSC cultures treated and not treated with AASs for 7 or 14 days, they were washed twice with cold PBS. Then, total protein from all cultured cells was extracted using radioimmunoprecipitation assay buffer (RIPA; Thermo Fisher Scientific Inc.) with the addition of a protease inhibitor cocktail (Sigma-Aldrich, Merck). Subsequently, cell lysates were sonicated and centrifuged at 10,000× *g* for 20 min at 4 °C. The supernatant was collected and stored at −20 °C. The protein concentration was measured using the DC^TM^ Protein Assay (Bio-Rad Protein Assay; Bio-Rad Laboratories GmbH, München, Germany), and bovine serum albumin (BSA; Sigma-Aldrich, Merck) served as a standard. Aliquots of cell lysates containing 30 or 20 µg of protein were solubilized in a Laemmli sample buffer consisting of 65.8 mM Tris-HCl pH 6.8, 2.1% SDS, 26.3% (*w*/*v*) glycerol, 0.01% bromophenol blue, and 5% (*v*/*v*) 2-mercaptoethanol (Bio-Rad Laboratories) and denatured at 99.9 °C for 3 min. After denaturation, the samples were separated via 10% SDS-PAGE under reducing conditions and electrotransferred onto a poly(vinylidene fluoride) (PVDF) membrane using a wet blotter in Genie Transfer Buffer (20 mM Tris, 150 mM glycine in 20% methanol, pH 8.4) for 90 min at a constant amperage of 350 mA. Then, the membranes were blocked with 5% non-fat dry milk in TBST (Tris-buffered saline with 0.1% *v*/*v* Tween20; Bioshop Inc., Burlington, VT, Canada) for 30 min at RT with gentle shaking followed by treatment (overnight at ~+4 °C) with the appropriate primary antibodies diluted in TBST (the type of antibodies, dilutions used, and manufacturer are given in Table 1, WB dilition). β-Actin served as a loading control (monoclonal mouse anti-β-actin, diluted 1:2000; Sigma-Aldrich, Merck). The membranes were washed and incubated with the goat anti-rabbit IgG horseradish peroxidase (HRP)-conjugated secondary antibody (Vector Laboratories; diluted 1:1000) for 1 h at RT. Immunoreactive protein bands were detected by chemiluminescence using Clarity™ Western ECL Blotting Substrate (Bio-Rad Laboratories). The blots were visualized using the ChemiDoc™, and all bands were quantified using the Image Lab™ 2.0 Software (Bio-Rad Laboratories). Semi-quantitative analysis was performed for three separately repeated experiments for each control and experimental group.

### 4.5. Total RNA Isolation and cDNA Synthesis

Total RNA was extracted from both poPSCs treated with AASs for 7 or 14 days and poPSCs non-treated with steroids. Total cellular RNA was isolated using the EZ-10 Spin Column Total RNA Mini Preps Super Kit (Bio Basic Canada Inc., Markham, ON, Canada) following the manufacturer’s protocol. The RNA quantity and quality were ascertained by the A260/A280 ratio using the NanoDrop ND2000 Spectrophotometer (Thermo Fisher Scientific Inc., Wilmington, DE, USA). Moreover, the RNA integrity was evaluated through the observation of 18S and 28S ribosomal bands after electrophoresis on 1% formaldehyde-agarose gel. The total RNA samples were stored frozen at −80 °C. First-strand cDNA was prepared by reverse transcription (RT) using 1 µg of total RNA, random primers, and a High-Capacity cDNA Reverse Transcription Kit (Applied Biosystems, Foster City, CA, USA) according to the manufacturer’s protocol. The 20 µL total reaction volume contained random primers, dNTP mix, RNAse inhibitor, and Multi Scribe Reverse Transcriptase. RT was performed in a T100 Thermal Cycler (Bio-Rad, Hercules, CA, USA) according to the following thermal profile: (1) 25 °C for 10 min, (2) 37 °C for 120 min, and (3) 85 °C for 5 min. Genomic DNA amplification contamination was checked using control experiments in which reverse transcriptase was omitted during the RT step. The samples were kept at −20 °C until further analysis.

### 4.6. Quantitative Real-Time qPCR

The real-time PCR was performed according to the manufacturer’s protocol. For quantitative analysis, the mRNA levels of the investigated genes: NFKBIA, RELA, NFKB1, and SLC39A9 were measured by RT-qPCR reactions for each sample using a reaction mix prepared as follows: 1× SYBR Select Master Mix (Thermo Fisher Scientific Inc.), 2 μL of forward and reverse primers (1 μM each), and 4 μL of 20× diluted cDNA in a final volume of 15 μL. A no-RT control run was conducted with DNase-digested RNA to verify that the digestion was successful and sufficient for the selected samples. The amplification protocol included an initial preheating at 50 °C for 2 min, initial denaturation at 95 °C for 10 min and 40 cycles of amplification (15 s at 95 °C and 60 s at 60 °C). A melting curve analysis was achieved at the end of each run. The RT-qPCR was carried out with a CFX96 Touch Real-Time PCR Detection System (Bio-Rad). The sequences of all the RT-qPCR primers are presented in Table 2. Alterations in the quantitative profiles (i.e., relative abundances; RAs) of relevant mRNA transcripts that were triggered by the exposure of in vitro cultured poPSCs to the selected AASs were rendered as the ratio of the target gene versus the reference GAPDH gene (coding for glyceraldehyde-3-phosphate dehydrogenase) in relation to expression in control samples (relative quantification, RQ = 1) using the 2^−ΔΔCt^ method [64].

### 4.7. ELISA Assays

Briefly, after the termination of both poPSC cultures treated and not treated with steroids for 7 or 14 days, they were washed twice with cold PBS. Then, total protein from all cultured cells was extracted similarly, as described above in [Sec sec4dot4-ijms-25-02833]. The concentration of NF-κB1 and NF-κB1 complex was determined in total protein samples by immunoassay, commercially available ELISA kit was used: Porcine Nuclear Factor Kappa B (NF-κB) ELISA Kit (cat. No. EIA06109p, QnD Wuhan Enlibio Biotech Co., Ltd., Wuhan, China). All samples were run in triplicate in the same assay. Assays were performed following the manufacturer’s instructions. Briefly, after protein extraction from poPSCs, 100 µL samples (containing the same amount of total protein) were added into 96-well plates and incubated for 90 min at +37 °C. Then, plates were washed twice in wash buffer (WBff) and incubated with biotinylated antibodies for 60 min at +37 °C. In the next step, plates were washed three times in WBff and incubated with a conjugate of horseradish peroxidase-avidin (HRP-avidin) in a working solution for 30 min at +37 °C. After incubation, plates were washed five times in WBff, a substrate for HRP-avidin was added and plates were incubated at +37 °C for up to 30 min. When the colouration of the highest standards became darker, and the colour gradient appeared, the incubation was stopped by adding Color Reagent C—1M sulfuric acid. The absorbance was read at 450 nm immediately in an LT-4500 microplate reader (LabTech, Winchester, UK). Results were analysed using the Four Parameter Logistic Curve as a part of Bioassay Data Analysis Tools on MyAssays Online Website (https://www.myassays.com/index.html; accessed on 4 September 2023).

### 4.8. Statistical Analysis

Statistical analysis was performed using Statistica 13 software (StatSoft, Inc.; Tulsa, OK, USA). For all the cell culture groups/variants, experiments were performed in triplicate (*n* = 3). Levene’s test for homogeneity of variance, the Shapiro–Wilk test for normality, and one-way ANOVA followed by the Dunnett post-hoc test were used to assess differences between control and experimental cultures. Western blot and real-time PCR analyses were repeated three times. The data are expressed as the mean ± SD. Statistical significance was established at * *p* ≤ 0.05, ** *p* ≤ 0.01, and *** *p* ≤ 0.001.

## 5. Conclusions

The ability of the NF-κB signalling pathway to inhibit apoptosis, induce proliferation, and enhance the angiogenesis process suggests that the NF-κB pathway may be an extremely important factor in the processes of carcinogenesis and tumour progression, especially when the component proteins of this pathway are located in the nucleus/cytoplasm and continuously activated. Anabolic steroids, including boldenone and nandrolone, are probably classified as carcinogenic compounds. Therefore, our study aimed to check whether boldenone and nandrolone can activate the NF-κB signalling pathway. However, we already knew that androgen receptors may be involved in the action of anabolic steroids. Therefore, this time we decided to investigate their effect on the ZIP-9 membrane androgen receptors.

**Figure 6:**
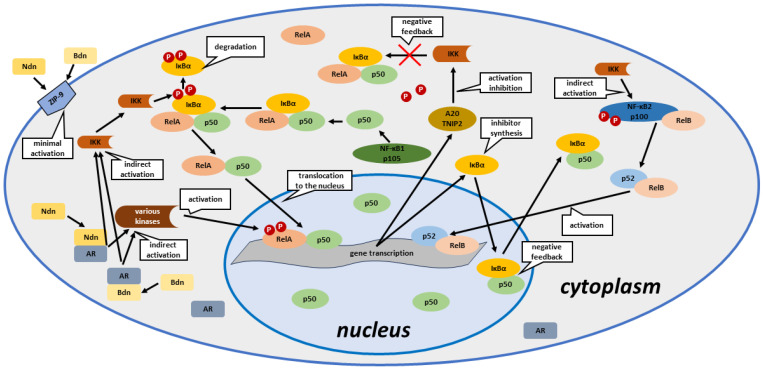
Simplified scheme of AAS-triggered activation of the NF-κB pathway and its regulation by negative feedback in poPSCs. **Ndn**—nandrolone; **Bdn**—boldenone; **ZIP-9**—membrane androgen receptor (Zrt- and Irt-like protein 9); **IKK**—IκB kinase; **AR**—androgen receptor; **IκBα**—nuclear factor of κ light polypeptide gene enhancer in B-cells inhibitor, α; **RelA**—transcription factor p65; **RelB**—transcription factor RelB; **NF-κB1 p105**—nuclear factor NF-κB p105 subunit; **NF-κB2 p100**—nuclear factor NF-κB p100 subunit; **p50**—NF-κB p50 subunit; **p52**—NF-κB p52 subunit; **A20/TNIP2**—A20/tumour necrosis factor (TNF)-interacting protein 2; **P**—phosphorylation; **various kinases**—e.g., Akt, PI3K.

It appears that the anabolic steroids we tested (in particular Ndn) can activate the NF-κB pathway by inducing phosphorylation of the RelA transcription factor and IκBα pathway-related inhibitory proteins. The NF-κB signalling pathway is regulated mainly by negative feedback, as evidenced by decreases in the expression of genes and unphosphorylated proteins of this pathway. Unfortunately, continuous exposure of poPSCs to anabolic steroids probably leads to cyclical activation of the NF-κB pathway and thus increases the risk of neoplastic transformation. Reduced expression of ZIP-9 at both gene and protein levels indicates that it does not play a significant role in signal transduction by anabolic steroids and that ZIP-9 is not indispensable for the activation of the NF-κB pathway. The diagram depicting the NF-κB-related pathway and possible routes of its activation by the selected AASs (Ndn or Bdn) are presented in Figure 6.

Finally, unveiling and insightfully interpreting Ndn- or Bdn-prompted intracellular molecular networks, inter-transcriptomic crosstalk, and cross-proteomic communication, which seem to be responsible for the oncogenic transformation of porcine ovarian putative stem cells, might expedite the elaboration of criteria pinpointed for the following: (1) negative selection of neoplastic cell derivatives of poPSCs; and (2) positive qualification of cytophysiologically healthy, self-renewable, and highly reprogrammable poPSCs for a broad spectrum of research areas. The latter encompasses not only modern assisted reproductive technologies (ARTs) such as somatic cell cloning (poPSCs as a source of excellent-quality nuclear donor cells) and in vitro embryo production (co-culture of ex vivo-matured nuclear recipient oocytes and ex vivo-fertilized or cloned embryos with poPSCs providing feeder monolayers) but also regenerative medicine (reconstructive surgery of female gonadal tissues with poPSC-based auto-, iso-, allo- or xenotransplants in patients afflicted with a variety of ovarian disorders or dysfunctions, e.g., polycystic ovary syndrome, premature menopause also designated as premature ovarian failure/insufficiency or benign and malignant/metastatic ovary-specific tumours).

## Figures and Tables

**Table 1:** List of primary antibodies used for immunofluorescence and Western blotting.

Antigen	ICC Dilution in PBST	WB Dilution in TBST	#cat.	Host/Clonality	Vendor
**NF-κB p65** **phosphorylated form**	1:100	1:1000	ab76302	Rabbit monoclonal	Abcam
**NF-κB p65 total form**	1:100	1:1000	ab32536		
**NF-κB p105**	1:100	1:1000	ab32360		
**IKBα** **phosphorylated form**	1:100	1:1000	ab133462		
**IKBα total form**	1:100	1:1000	ab32518		
**ZIP-9**	Not applicable	1:500	SAB3500599	Rabbit polyclonal	Sigma-Aldrich, Merck

**Table 2:** Primers used for RT-qPCR.

Gene (*Sus Scrofa Domesticus*)	F/R	Primer Sequence (5′→3′)	T_m_ (°C)	RefSeq Accession Number (NCBI Nucleotide Database)	Reference
***NFKBIA***	F	TGTGATCCTGAGCTCCGAGACTTT	57.4	NM_001005150	[65]
	R	TTGTAGTTGGTGGCCTGCAGAATG	57.4		
***RELA***	F	ACATGGACTTCTCAGCCCTTCTGA	57.4	NM_001114281	
	R	CCGAAGACATCACCCAAAGATGCT	57.4		
***NFKB1***	F	CCCATGTAGACAGCACCACCTATGAT	59.5	NM_001048232	
	R	ACAGAGGCTCAAAGTTCTCCACCA	57.4		
***SLC39A9***	F	TGTTACGTGGCTGGAATCATTC	53.0	no data	Primers designed for this work
	R	CATGTTCATGGGCAACTGGTAT	53.0		
***GAPDH***	F	CCCACGAGCACACCTCAGAA	55.9	NM_001206359	[66]
	R	TGCAGCCTGTACTCCCGCT	55.4		

## Data Availability

Data are contained within the article.

## Abbreviations

A20/TNIP2	A20/tumour necrosis factor (TNF)-interacting protein 2
AASs	Anabolic androgenic steroids
Akt	A member of serine/threonine-specific protein kinase family (also known as protein kinase B; PKB) that plays a pivotal function in controlling the molecular balance between survival and death pathways in cells
AR	Androgen receptor
ASCs	Adult stem cells
ATF-1	Cyclic AMP-dependent transcription factor ATF-1
Bdn	Boldenone
*BIRC2*	Baculoviral IAP repeat-containing protein 2
cAMP	Cyclic adenosine monophosphate
*CFLAR*	CASP8 and FADD-like apoptosis regulator
COX-2	Cyclooxygenase-2
CREB	cAMP response element-binding protein
CSCs	Cancer stem cells
ENG	Endoglin
ERK1/2	Extracellular signal-regulated protein kinases 1 and 2; also known as p44 mitogen-activated protein (MAP) kinase (a 44-kDa isoform of MAPK) and p42 mitogen-activated protein (MAP) kinase (a 42-kDa isoform of MAPK), respectively
ESCs	Embryonic stem cells
FSCs	Fetal stem cells
HUVEC	Human umbilical vein endothelial cells
HRP	Horseradish peroxidase
IARC	International Agency for Research on Cancer)
ICC	Immunocytochemistry
IκBα	Nuclear factor of κ light polypeptide gene enhancer in B-cells inhibitor, α
IκBβ	Nuclear factor of κ light polypeptide gene enhancer in B-cells inhibitor, β
IKK	IκB kinase
IL-4	Interleukin 4
*ITGB1*	Integrin β1
mAR	Membrane androgen receptors
MSCs	Mesenchymal stem cells
Ndn	Nandrolone
NF-κB1	Nuclear factor NF-κB p105 subunit
NF-κB2	Nuclear factor NF-κB p100 subunit
NF-κB	Nuclear factor-κB (nuclear factor κ-light-chain-enhancer of activated B cells); a pleiotropic inducible transcription factor that occurs in almost all cell types and is the endpoint of a series of signal transduction events that are initiated by a vast array of stimuli related to many biological processes such as cytodifferentiation, cell growth, tumorigenesis, apoptosis, inflammation, and immunity
NLS	Nuclear localization sequence
PI3K	Phosphatidylinositol 3-kinase; a downstream kinase activated by receptor tyrosine kinases that generates a series of phosphorylated phosphoinositides, which recruit 3-phosphoinositide-dependent protein kinase-1 (PDPK1) activity to the plasma membrane, leading to activation of Akt
poPSCs	Porcine ovarian putative stem cells
PSCs	Putative stem cells
Rel	c-Rel; protein encoded by a protooncogene REL; represents a subunit of a homo- or heterodimeric complex designated as NF-κB and, simultaneously, exhibits the properties of DNA-binding transcription factor activity and chromatin binding; etymology of the name assigned to this protein stems from the involvement of Rel in the etiopathogenesis of the avian viral disease known as reticuloendotheliosis associated with the development of B- and T-cell lymphomas; a protein belonging to the Rel homology domain/immunoglobulin-like fold, plexin, transcription factor (RHD/IPT) family of ubiquitous NF-κB transcription factors, whose members regulate genes engaged in apoptosis, inflammation, the immune response, and oncogenic processes
RelA	Transcription factor p65
RelB	Transcription factor RelB
ROS	Reactive oxygen species
SCs	Stem cells
SD	Standard deviation
SSEA-4	Stage-specific embryonic antigen-4
STAT6	Signal transducer and activator of transcription 6
TAD	C-terminal transactivation domain
THY	Thymocyte differentiation antigen
WB	Western blot
WBff	Wash buffer
ZIP-9	Zinc transporter member 9; also designated as solute carrier family 39 member 9 (SLC39A9) or transmembrane zinc-influx transporter (Zrt)- and transmembrane iron-influx transporter (Irt)-like protein (ZIP) 9; Zrt- and Irt-like protein 9; represents both zinc (Zn^2+^)-iron (Fe^2+^) permease (ZIP) family and a novel membrane androgen receptor (mAR) family related to the extranuclear action of androgens
